# Interrupted incubation: How dabbling ducks respond when flushed from the nest

**DOI:** 10.1002/ece3.7245

**Published:** 2021-02-23

**Authors:** Rebecca Croston, C. Alex Hartman, Mark P. Herzog, Sarah H. Peterson, Jeffrey D. Kohl, Cory T. Overton, Cliff L. Feldheim, Michael L. Casazza, Joshua T. Ackerman

**Affiliations:** ^1^ U.S. Geological Survey Western Ecological Research Center Dixon CA USA; ^2^ California Department of Water Resources Suisun Marsh Program West Sacramento CA USA

**Keywords:** dabbling duck, disturbance, gadwall, GPS–GSM transmitter, iButton, incubation recess, investigator effects, mallard, nest break, recess duration, recess timing

## Abstract

Nesting birds must provide a thermal environment sufficient for egg development while also meeting self‐maintenance needs. Many birds, particularly those with uniparental incubation, achieve this balance through periodic incubation recesses, during which foraging and other self‐maintenance activities can occur. However, incubating birds may experience disturbances such as predator or human activity which interrupt natural incubation patterns by compelling them to leave the nest. We characterized incubating mallard *Anas platyrhynchos* and gadwall *Mareca strepera* hens’ responses when flushed by predators and investigators in Suisun Marsh, California, USA. Diurnal incubation recesses initiated by investigators approaching nests were 63% longer than natural diurnal incubation recesses initiated by the hen (geometric mean: 226.77 min versus 142.04 min). Nocturnal incubation recesses, many of which were likely the result of predators flushing hens, were of similar duration regardless of whether the nest was partially depredated during the event (115.33 [101.01;131.68] minutes) or not (119.62 [111.96;127.82] minutes), yet were 16% shorter than natural diurnal incubation recesses. Hens moved further from the nest during natural diurnal recesses or investigator‐initiated recesses than during nocturnal recesses, and the proportion of hen locations recorded in wetland versus upland habitat during recesses varied with recess type (model‐predicted means: natural diurnal recess 0.77; investigator‐initiated recess 0.82; nocturnal recess 0.31). Hens were more likely to take a natural recess following an investigator‐initiated recess earlier that same day than following a natural recess earlier that same day, and natural recesses that followed an investigator‐initiated recess were longer than natural recesses that followed an earlier natural recess, suggesting that hens may not fulfill all of their physiological needs during investigator‐initiated recesses. We found no evidence that the duration of investigator‐initiated recesses was influenced by repeated visits to the nest, whether by predators or by investigators, and trapping and handling the hen did not affect investigator‐initiated recess duration unless the hen was also fitted with a backpack‐harness style GPS–GSM transmitter at the time of capture. Hens that were captured and fitted with GPS–GSM transmitters took recesses that were 26% longer than recesses during which a hen was captured but a GPS–GSM transmitter was not attached. Incubation interruptions had measurable but limited and specific effects on hen behavior.

## Graphical abstract

We characterized incubating mallard *Anas platyrhynchos* and gadwall *Mareca strepera* hens’ responses when flushed by predators and investigators. Our results suggest that hens behave differently during recesses initiated by hens versus investigators or predators, and during diurnal versus nocturnal recesses, as measured by their time away from the nest and their locations on the landscape during different types of recesses.

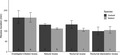

## 1 INTRODUCTION

In order to nest successfully, incubating birds must balance their own metabolic needs with maintaining the proper physical environment for egg development (Afton & Paulus, 1992). Many birds, particularly those with uniparental incubation, manage these competing needs by taking periodic breaks from incubation to perform self‐maintenance activities such as foraging and preening. Therefore, natural incubation patterns (e.g. the timing and duration of absences from the nest) reflect this balance of competing needs (Reid et al., 2002; Tinbergen & Williams, 2002). However, incubating birds may experience interruptions to natural incubation patterns from the approach of predators or other animals, which force them to leave their nests at times that they would otherwise incubate. Interruptions may affect incubation constancy and the timing of incubation recesses, which may influence nest success through energetic costs associated with reheating eggs (Tinbergen & Williams, 2002; Williams, 1996), exposure to thermodynamic stress (Mougeot et al., 2014), altered risk of depredation (Olsen and Rohwer 1998, Stein & Ims, 2016; reviewed in Götmark, 1992), or changes in overall incubation period length (Hepp et al., 2006; Samelius & Alisauskas, 2001; Carter et al., 2014). Thus, how nesting birds respond to interruptions may also play an important role in determining nest success.

Incubating hens may behave differently during an incubation recess that was triggered by an interruption than during a recess that they initiated themselves. During natural incubation recesses, hens perform self‐maintenance behaviors such as foraging and preening, but when an incubation recess is involuntary, e.g. due to an approaching predator, hens may forego self‐maintenance behaviors to increase vigilance (Henson & Grant, 1991) or perform other behaviors not typically observed during natural incubation recesses. Hens may alter the amount of time that they take to return to the nest (Henson & Grant, 1991; Mougeot et al., 2014), how far they travel from the nest, and how they use the landscape during the incubation recess. Effects of interruptions on nest attendance and incubation recess timing may also vary with the time of day that interruptions occur (Livezy, 1980; Gloutney et al., 1993), with nest age (Garrettson et al., 2010), or with repeated interruptions (Conomy et al., 1998; Baudains & Lloyd, 2007).

Predators often flush waterfowl hens from nests during partial depredation events (Ackerman, Eadie, Yarris, et al., 2003; Ackerman & Eadie, 2003; Forbes et al., 1994) and other attempted depredation events (Croston, Ackerman, et al., 2018). Yet, many times when predators visit a nest, the remaining eggs are still viable and can produce ducklings (e.g. 27% of partially depredated mallard nests still successfully produced ducklings; Ackerman, Eadie, Loughman, et al., 2003). Therefore, understanding hen incubation behavior during predator visits can elucidate mechanisms contributing to reproductive success. In addition, studies of nesting ecology typically require investigators to make periodic visits to nests to collect data and might interrupt natural incubation recess timing. By visiting nests and/or capturing birds at the nest, investigators may alter bird behavior, particularly when birds are captured and handled or are fitted with monitoring equipment (reviewed in Lameris & Kleyheeg, 2017). Hensc (e.g. Melzack et al., 1959) may also become habituated or sensitized to repeated approaches to the nest (Baudains & Lloyd, 2007; Vennesland, 2010). Understanding hen responses to these events would more fully describe the impacts of incubation interruptions on reproductive success and could improve investigator methodology.

Here we investigated the duration of natural diurnal incubation recesses, nocturnal incubation recesses, many of which were likely initiated by predators, and diurnal incubation recesses initiated by investigators for mallard (*Anas platyrhynchos*) and gadwall (*Mareca strepera*). We also examined variation in hens’ landscape use during incubation recesses among the three types of incubation recesses, and the effects of repeated nest visits and of capturing hens on incubation recess frequency and duration. Data supporting this manuscript are available as a USGS Data Release (Croston et al., 2021).

## 2 METHODS

### 2.1 Nest searching, monitoring, and hen capture

We monitored mallard and gadwall nests at the Grizzly Island Wildlife Area, Suisun Marsh, CA, USA during the 2015–2018 breeding seasons. We located nests by systematically searching through upland fields every 3 weeks following standard nest searching methods modified from McLandress et al. (1996). To initially locate nests, we flushed incubating hens by dragging a 50‐m rope strung between two slow‐moving all‐terrain vehicles (ATVs) across the tops of vegetation. When a hen flushed, we systematically searched the area until we located the nest. We marked nests with a 2‐m bamboo stake placed 4 m north of the nest, and a stake placed at vegetation height on the south edge of the nest bowl. We candled eggs (*sensu* Weller, 1956) to determine the incubation day (number of days since clutch completion) on which the nest was found. For nests found during egg laying, we estimated the date of clutch completion by counting forward to the total clutch size as determined on a later visit, and assuming that one egg was laid per nest‐day. For nests found after egg laying had concluded, we subtracted the average incubation day of all eggs on the first visit from the date of the first visit to estimate the date of clutch completion.

We returned to each nest weekly until the nest either hatched or failed. We approached nests and recorded the time of our approach and whether the hen was present and flushed from the nest. We candled eggs during each visit to monitor embryonic development and to confirm nest status. After each visit, we re‐covered eggs with nesting material, imitating typical hen behavior at the onset of an incubation recess. Nest visits typically lasted approximately 10 min, unless a hen was captured (see below), in which case processing the hen took up to 20 min. Initial nest visits (nest discovery) were also longer (~20 min), but these were excluded from all analyses.

We attempted to capture hens with hand‐held long‐handled dip nets during regular weekly nest visits once the eggs were projected to be ≥8 days into incubation (as hens are less likely to abandon older nests; Ackerman, Eadie, Yarris, et al., 2003; Ackerman & Eadie, 2003). A capture attempt consisted of approaching a marked nest rapidly on foot from within 5 m and swinging the dip net over the nest's known location in order to catch the hen as she flushed. Once captured, we measured hen mass, flattened wing chord length, short tarsus length, and exposed culmen length (Dzubin & Cooch, 1992) for use in a related study. Some hens were also fitted with backpack‐harness style Global Positioning System–Global System for Mobile Communications (GPS–GSM) transmitters (see below).

### 2.2 Hen movements during incubation recesses

We affixed one of three types of solar‐powered GPS–GSM transmitter to 76 hens using an adjustable body harness (Dwyer, 1992) made of high‐grade braided flat automotive elastic (Conrad‐Jarvis, Corp.). We used Ecotone Saker L series transmitters (Ecotone Telemetry, Sopot, Pomerania Province, Poland; weight 17 g) in 2015–2018., Ecotone Crex series transmitters (Ecotone Telemetry; weight 14 g) in 2017, and Ornitela Ornitrack‐15 transmitters (Ornitela UAB, Vilnius, Lithuania; weight 15 g; transmitters did not exceed 3% of the hens’ body weight) in 2018. All transmitters collected GPS locational data at a frequency ranging from every 15 min to every 6 hr depending on transmitter battery levels. Transmitters utilized 2G/3G cellular networks to transmit coded data to the manufacturer who provided decoded and quality‐controlled data via web‐interface. Hen locations during incubation recesses were later determined to be within either upland or wetland habitat based on aerial imagery and land management patterns.

### 2.3 Incubation recess detection and categorization

We used nest temperature data to determine whether hens were present or absent from nests throughout the day and night. During the initial nest visit, we placed two iButton temperature dataloggers (Model DS1922L‐F5#, Maxim Integrated Products, Inc.) at each nest, one in the center of the nest bowl, flush with the apical surface of the eggs, and the second immediately south of the rim of the nest bowl in order to record local ambient temperature. Prior to deployment, we programmed iButtons to record temperature at intervals of either 4 (2015) or 8 min (2016–2018). Each iButton was fitted within but protruding slightly from the top of a cream‐colored rubber stopper, which was itself fitted to the top of a long nail and anchored firmly in the ground. In 2015 we replaced iButtons in nests every 2 weeks to prevent on‐board memory becoming full. For analysis, we censored 2015 data to 8‐min intervals for direct comparison with the 2016–2018 data.

We identified hen presence and absence from the nest using monotonic changes in nest temperature relative to each nest's own daily variation in temperature, following methods described in Croston, Hartman, et al. (2018). From these data, we derived the start time, end time, and duration of each incubation recess. We categorized incubation recesses based on why and when they were initiated as one of 4 types: (a) a natural recess occurred when an incubation recess was initiated during the day between 04:00 and 21:00 (Croston et al., 2020). Previously, through continuous video monitoring, we determined that recesses during this time most likely represent natural incubation recesses initiated by the hen (Croston, Hartman, et al., 2018). In some of these instances, predators may have flushed hens from nests, as we could not distinguish diurnal predation events from natural recesses. However, the vast majority of nest depredation events (>80%) within this system occur at night (Croston, Ackerman, et al., 2018), suggesting that relatively few daytime recesses were predator‐initiated. (b) An investigator‐initiated recess occurred when an incubation recess was initiated by investigators approaching the nest and flushing the hen. We associated nest temperature data during investigator‐initiated recesses with the actual timing of our nest visits by matching each nest visit time with the nearest recess that was recorded with automated recess detection, if that recess occurred within 60 min of our arrival at the nest. The 60‐min window served only to align known investigator‐recorded visit times with the correct iButton‐derived recess start times, compensating for lag in recess detection and any discrepancy due to timekeeping in the field. Approximately 84% of the recesses that were aligned within this 60‐min window were detected within 15 min of the actual investigator‐recorded nest visit time. If there was not an absence from the nest recorded in the iButton data within 60 min of our arrival, we considered this a “failure to detect a nest recess” and excluded that nest visit from the dataset. We also excluded data associated with nest visits when the hen was not present when we arrived at the nest, because in these cases we could not determine if the hen flushed undetected or was taking a natural incubation recess. (c) A nocturnal recess occurred when an incubation recess was initiated at night, between 21:00 and 04:00, but was not accompanied by evidence of a nest depredation at the subsequent nest visit. These recesses likely were associated with predator activity near the nest, as investigators never visited nests between 21:00 and 04:00 and recesses rarely occurred at this time (Croston et al., 2020), yet almost all mammalian depredation of duck nests occurred at this time (Croston, Ackerman, et al., 2018). (d) A nocturnal depredation recess occurred when an incubation recess was initiated at night between 21:00 and 04:00, and during the subsequent nest visit investigators found broken eggshells and/or missing eggs indicative of a depredation event. Multiple nocturnal recesses occurring within a single nest‐week in which evidence of depredation was found were all considered nocturnal depredation recesses, because continuous video monitoring of a subset of nests showed that depredation events were equally likely to occur during the first, middle, last, or over multiple nocturnal recesses within a nest‐week (R. Croston *unpub.data*), and nocturnal depredation recesses that were the only one in a nest‐week did not differ in duration from nocturnal depredation recesses that were one of multiple within a nest‐week (Welch's *t*‐test; *p* = .66); therefore, we could not yet identify which of multiple nocturnal recesses were depredation events with these data.

For all nests, we excluded data collected (a) on and prior to the clutch completion date, because irregular incubation during egg laying makes automated recess detection less reliable, (b) on and after the date the nest was determined to be no longer active (nest either hatched, was abandoned, or was completely depredated), (c) on days that iButtons were initially placed in nests, as iButtons could not have recorded the time that hens left the nest during initial nest visits, and (d) from nests on days that we searched for nests within the same nesting field. During nest searches, investigators searched fields for a longer period of time, and hens may have been unwilling to return to nests while investigators remained nearby.

### 2.4 Statistical analysis

#### 2.4.1 Recess duration among recess types

To test for differences in incubation recess duration among recess types, we fit a linear mixed model (LMM) predicting recess duration using R package *lme4* (Bates et. al, 2019). We included type of recess (natural, investigator‐initiated, nocturnal, nocturnal depredation) as a fixed effect in this model, while also controlling for incubation day, species, and time of day. By definition, the type of recess is confounded with the time of day. To address this, we set time of day as the number of minutes elapsed since the start of the day (04:00) for diurnal recesses or the number of minutes elapsed since the start of night (21:00) for nocturnal recesses. We allowed for a three‐way interaction between type of recess, species, and time of day as the timing of incubation recesses differs with time of day uniquely for mallard and gadwall (Croston et al., 2020). We also included ambient temperature at the nest at the start of the recess and day of year as fixed effects, as both influence recess duration (Croston et al., 2020). We included nest identification as a random effect. In this and all subsequent models, recess duration was log‐transformed to improve normality. For this model, we excluded investigator‐initiated recesses if the hen was flushed early or incidentally by the approach of an ATV (such that all investigator‐initiated flushes were from foot), and we excluded data collected during or after hens were successfully trapped, as trapping and handling may influence the duration of a hen's absence from the nest (we assessed trapping effects separately below).

We fitted this and all subsequent LMMs with restricted maximum likelihood, and with Type III Wald *F*‐tests and Kenward‐Roger degrees of freedom (R package *car*, Fox & Weisberg, 2019). We present summary results both as raw data and as the median results of 1,000 bootstrapped predictions, and 95% prediction intervals which reflect the 2.5th and 97.5th quantile of the bootstraps (Gelman & Hill, 2007). For this and all models, bootstrap predictions were made at the means of each non‐focal parameter: for this model incubation day 13, ambient temperature 24°C, day of year 145. Because each recess type occurred at a different time of day with little overlap among recess types, we elected to predict each recess type at the mode hour of occurrence for that recess type (natural 16:00, investigator‐initiated 09:00, nocturnal 23:00, nocturnal depredation 23:00). Thus, predicted differences by recess type necessarily include differences due to time of day, in addition to recess type.

#### 2.4.2 Maximum distance from the nest and habitat use during natural recesses versus recesses resulting from incubation interruption

We examined whether hens differed in the distance they travelled from the nest or the habitat they used among natural, nocturnal, nocturnal depredation, and investigator‐initiated recesses. We aligned hen location data with incubation recess times from iButton data and determined the maximum distance from the nest that each hen was recorded during each recess. We fit an LMM with the maximum distance from the nest during each recess as the response variable, and the interaction between time of day and type of recess as a fixed effect, while also accounting for duration of recess and incubation day. We included nest identification as a random effect.

To investigate differences in habitat use (upland versus wetland) during different types of recess, we calculated the proportion of locations for each recess in which hens were in wetland habitat. We then fit a generalized binomial mixed model predicting the proportion of locations per recess in which hens were recorded in wetland habitat. For this analysis, we combined nocturnal recesses with nocturnal depredation recesses due to relatively low sample sizes for these groups. We included the type of recess as a fixed effect and allowed it to interact with time of day, while controlling for duration of recess and incubation day. We also controlled for the frequency of data collection (number of locations recorded/time), as this varied throughout the study due to fluctuating battery levels. We included nest identification as a random effect. Bootstrap predictions were made at incubation day 13, 30 min data frequency, 240 min recess duration, and at the mode hour for each recess type (natural 16:00, investigator‐initiated 09:00, nocturnal 03:00). For both models, we excluded recesses for which only one location was recorded.

#### 2.4.3 Probability and duration of natural recesses following investigator‐initiated or natural recesses that same day

If an incubating hen that has been interrupted by investigators does not use that recess to meet self‐maintenance needs as it would with a natural recess, it may still require additional recesses later in that day. To investigate whether an investigator‐initiated recess earlier in the day influenced the probability of hens initiating recesses later in the day, we fit a generalized binomial mixed model predicting the probability of hens initiating a recess after a natural recess versus after an investigator‐initiated recess. We included the type of recess as a categorical predictor, and because mallard more often take two recesses per day, whereas gadwall more often take one recess per day (in the afternoon; Croston et al., 2020), we allowed a three‐way interaction between type of recess, time of day, and species. We also controlled for the number of recesses that occurred earlier on that same day, and included nest identification as a random effect. Bootstrap predictions were made at incubation day 13, ambient temperature 24°C, day of year 145, one recess prior to the current one on that day, and at the mode hour for each recess type (natural 16:00, investigator‐initiated 09:00). We excluded nest‐days with a nocturnal recess prior to a diurnal or investigator‐initiated recess because we do not know what effect, if any, being flushed from the nest overnight may have on the next days’ incubation recess timing.

We also investigated whether the duration of a natural recess that followed an investigator‐initiated recess was different from the duration of a natural recess that followed another natural recess. For nest‐days on which hens took at least two recesses (*N* = 3,340 days), we fit an LMM with natural log‐transformed duration of the second recess as the response variable and with a categorical fixed effect indicating whether the first recess was an investigator‐initiated recess or a natural recess (on a day that the nest was not visited). We allowed interactions of this categorical variable with species and time of day as fixed effects, and we controlled for effects of incubation day, ambient temperature, and day of year (Croston et al., 2020). We included nest identification as a random effect. Bootstrap predictions were made at incubation day 13, ambient temperature 24°C, and day of year 145. As above, we excluded nest‐days with a nocturnal recess prior to a diurnal or investigator‐initiated recess.

#### 2.4.4 Hen habituation and sensitization to repeated incubation interruptions

We examined potential changes in hen behavior after multiple visits by predators or humans (e.g., habituation or sensitization) using two measures of repeated incubation interruption as predictors: 1) the total number of investigator‐initiated recesses prior to the current investigator‐initiated recess (hereafter, cumulative visit number; the visit was not included in the count if the hen did not flush from the nest on our arrival) and 2) the number of times a hen had taken a recess at night prior to the current investigator‐initiated recess (hereafter; cumulative nocturnal flush number). We fit an LMM predicting the duration of investigator‐initiated recesses after repeated incubation interruption by either investigators or predators, which included cumulative visit number and the cumulative nocturnal flush number as predictors, while also controlling for time of day, ambient temperature, day of year, and incubation day, and nest identification as a random effect. Bootstrap predictions were made at incubation day 13, three prior nest visits, three prior nocturnal flushes, ambient temperature 24°C, day of year 145, and at the mode hour of 09:00 for investigator‐initiated recesses. We limited this analysis to nests that had not been partially depredated at the time of data collection, and we excluded nest visits during which hens were captured. We included only data collected on or earlier than incubation day 24 because nest visitation increased around day 24 in anticipation of hatch in order to measure and mark ducklings for a related study.

#### 2.4.5 Effects of hen trapping on incubation recess duration

Lastly, we evaluated the effects of hen trapping effort and hen trapping success on the natural log‐transformed duration of recesses during which we attempted to trap hens. This model included a categorical predictor describing investigator trapping effort and success for each visit as one of either: (a) no attempt at trapping—investigator approached the nest but made no effort to capture the hen; (b) trapping was attempted but was not successful—investigator attempted to capture the hen with a hand‐held net, but the attempt was not successful; (c) investigator successfully trapped the hen with a hand‐held net and processed her (banded, weighed, and measured) but did not fit her with a GPS–GSM transmitter; or (d) investigator successfully trapped the hen with a hand‐held net, processed her, and fitted her with a GPS–GSM transmitter. We allowed this category to interact with the time of day of our visit while also controlling for incubation day, and included nest identification as a random effect. Bootstrapped predictions were generated at incubation day 13 and at the mode hour of 09:00 for investigator‐initiated recesses.

## 3 RESULTS

We recorded a total of 14,218 incubation recesses across 8,954 nest‐days at 788 nests (438 mallard, 350 gadwall; Croston et al., 2021) between April and July 2015–2018.

### 3.1 Recess duration among recess types

**Figure 1:**
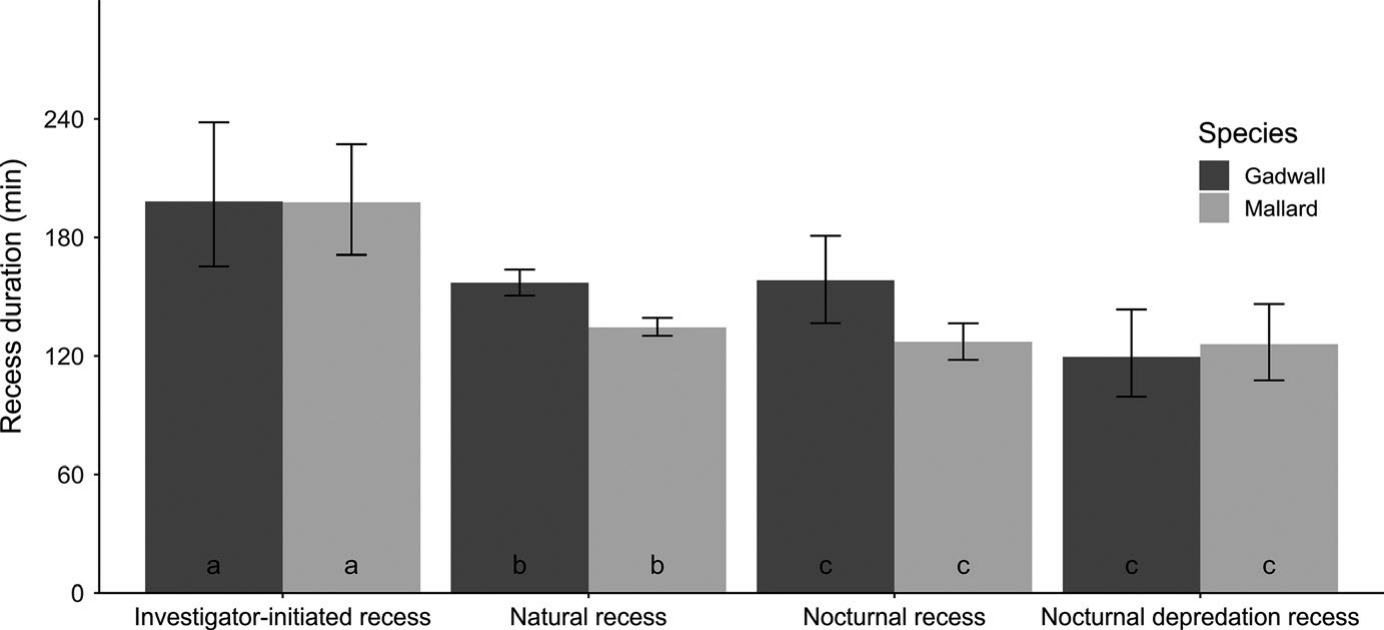
Predicted recess duration grouped by type of recess and species for gadwall and mallard at the Grizzly Island Wildlife Area, Suisun Marsh, CA, 2015–2018. Predictions are bootstrapped over 1,000 iterations from a linear mixed model (LMM) which included type of recess (natural, investigator‐initiated, nocturnal, nocturnal depredation), species, and their interaction with time of day as fixed effects. Ambient temperature and day of year were also included as fixed effects. Nest identification was included as a random effect. Bars represent 95% prediction intervals. Predictions were generated with time of day held to the mode hour of for each type of recess and all other non‐focal parameters held to their means. Bar labels represent significant differences—shared labels indicate no significant difference based on Tukey's post hoc comparisons

On average, natural recesses lasted 142.04 min [140.09; 144.03] (geometric mean [95% CI] from raw data) (*N* = 9,881). Nocturnal recesses lasted 119.62 [111.96; 127.82] minutes (*N* = 848). Nocturnal depredation recesses lasted 115.33 [101.01; 131.68] minutes (*N* = 247). Investigator‐initiated recesses lasted 226.77 [212.86; 241.59] minutes (*N* = 424). Recess duration varied significantly among recess types, and the relationship between recess duration and type of recess also differed among species after accounting for their interaction with time of day (type of recess *species * time of day *F*_3,10,761.31_ = 4.55, *p* < .005; Figure 1), as well as for the effects of ambient temperature (*F*_1,10,639.24_ = 1,059.03, *p* < .0001), incubation day (*F*_1,4,044.70_ = 11.14, *p* < .005), and day of year (*F*_1,761.49_ = 51.95, *p* < .0001). Investigator‐initiated recesses were significantly longer than all other recess types, and there was no difference in recess duration between nocturnal recesses and nocturnal depredation recesses (Figure 1).

### 3.2 Maximum distance from the nest and habitat use during natural recesses versus recesses resulting from incubation interruption

**Figure 2:**
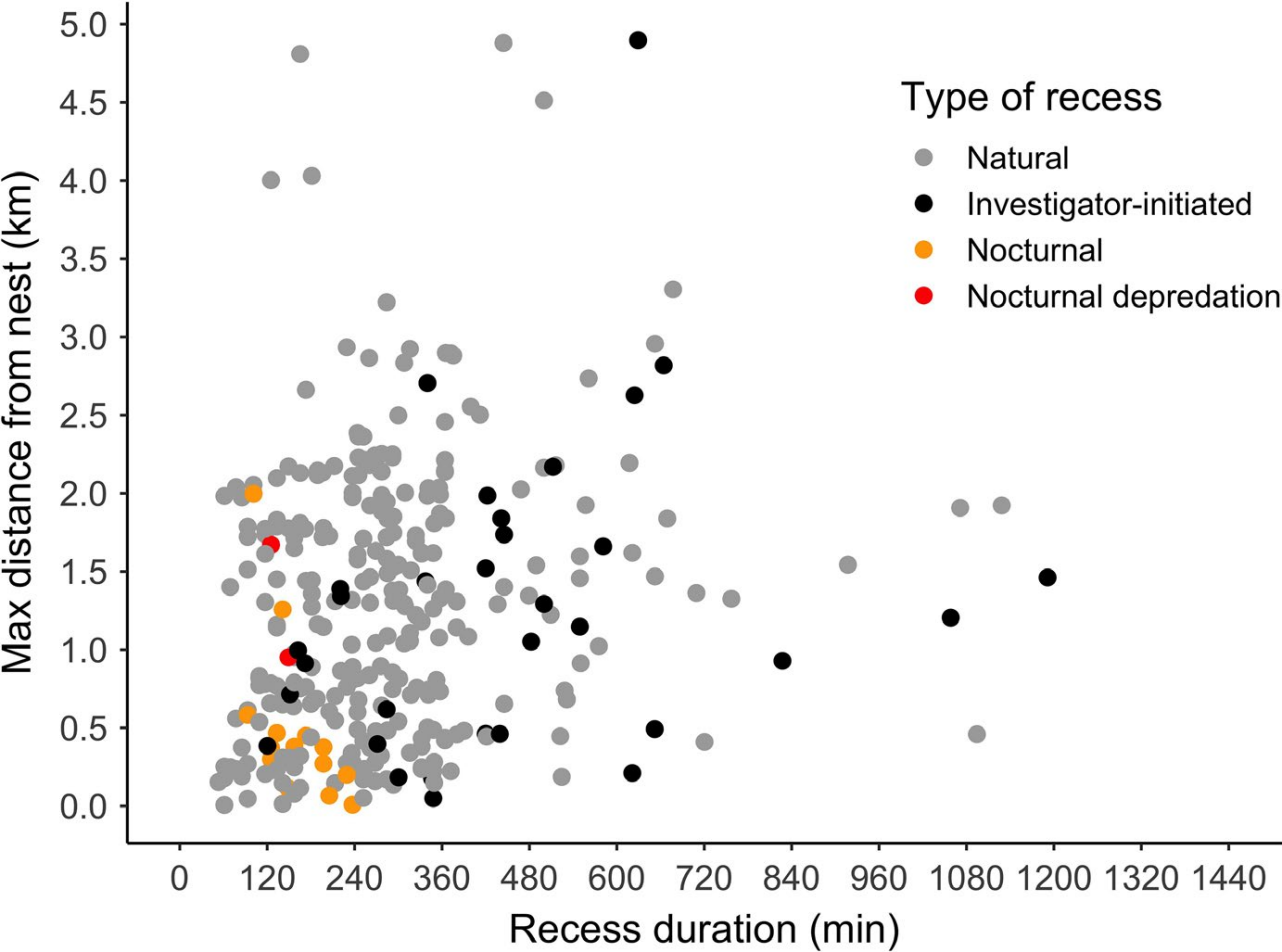
Maximum distance hens travelled from their nests during recesses by recess type, as a function of the duration of the recess, for gadwall and mallard hens nesting at the Grizzly Island Wildlife Area, Suisun Marsh, CA, 2015–2018. Two recesses (one natural and one investigator‐initiated) exceeded 1,440 min duration and were excluded for readability

We recorded 321 recesses among 71 nesting hens fitted with GPS‐GSM transmitters (4.52 ± 3.39 recesses per hen; mean ± *SD*). The mean maximum distance from the nest that transmittered hens were observed was 1.31 ± 0.88 km during natural recesses (*N* = 271), 1.28 ± 0.99 km during investigator‐initiated recesses (*N* = 33), 0.47 ± 0.51 km during nocturnal recesses (*N* = 15), and 1.31 ± 0.51 during nocturnal depredation recesses (*N* = 2). After holding all non‐focal parameters to their means, the mean maximum distance from the nest that transmittered hens were observed during natural recesses was 54% greater than during nocturnal recesses (1.28 versus. 0.83), and was 11% greater during investigator‐initiated recesses than during natural recesses (1.42 versus. 1.28; *F*_3,273.60_ = 3.07, *p* < .05) after accounting for time of day (*F*_1,310.37_ = 0.91, *p* = .34), incubation day (*F*_1,299.57_ = 0.39, *p = *.53), and recess duration (*F*_1,307.39_ = 1.61, *p* = .21; Figure 2).

The mean proportion of locations that occurred in wetland habitat was 0.70 ± 0.41 during natural recesses, 0.67 ± 0.41 during investigator‐initiated recesses, and 0.53 ± 0.44 during nocturnal recesses and nocturnal depredation recesses combined. When time of day was held to the mode hour for each recess type and all other non‐focal model parameters were held to their means, the predicted proportion of locations in wetland habitat was 0.77 [0.65; 0.86] during a natural recess, 0.82 [0.58; 0.94] during an investigator‐initiated recess, and 0.31 [0.15; 0.65] during a nocturnal recess. The odds of a hen being located in wetland habitat were 7.2 times greater during a natural recess than during a nocturnal recess and were 2.4 times greater during an investigator‐initiated recess than during a natural recess after accounting for time of day (type of recess * time of day χ^2^ = 228.07, *df* = 2, *p* < .0005), and after accounting for recess duration (χ^2^ = 4.68, *df* = 1, *p = *.03), data frequency (χ^2^ = 0.14, *df* = 1, *p = *.93), and incubation day (χ^2^ = 0.01, *df* = 1, *p = *.93).

### 3.3 Probability and duration of natural recesses following investigator‐initiated or natural recesses that same day

**Figure 3:**
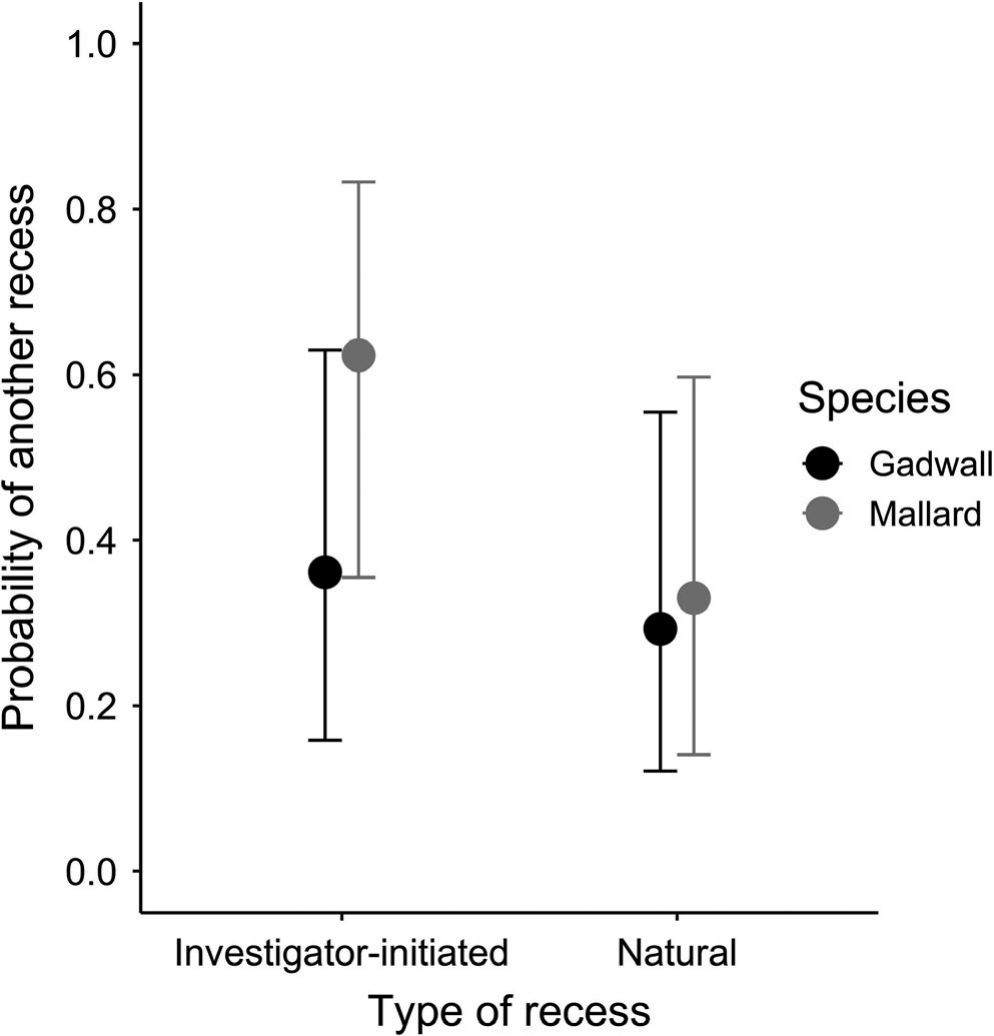
For mallard and gadwall hens at the Grizzly Island Wildlife Area, Suisun Marsh, CA, 2015–2018, the predicted probability of a hen taking a recess after the current one. Predictions are from a generalized linear mixed model (GLMM) which included the type of the current recess and its interaction with time of day and species, incubation day, and the number of recesses that occurred earlier in the day. Nest identification was included as a random effect.Predictions are bootstrapped over 1,000 iterations and shown at noon on the mean incubation day, with one previous recess. Bars represent 95% prediction intervals

Mallard hens were 29% more likely to initiate a recess later in the day if the current recess was initiated by investigators than if the current recess was initiated by the hen, whereas gadwall hens were 7% more likely to initiate a recess later in the day if the current recess was initiated by investigators than if the current recess was initiated by the hen (type of recess * species χ^2^ = 5.32, *p* < .05; Figure 3), after controlling for the number of previous recesses that day (χ^2^ = 34.71, *p* < .0001), incubation day (χ^2^ = 0.68, *p* = .41), the interaction between time of day and type of recess (χ^2^ = 1.47, *p* = .23) and the interaction between time of day and species (χ^2^ = 4.71, *p* < .05).

Natural recesses that followed an investigator‐initiated recess earlier in the same day (*N* = 120) were longer on average (173.50 [154.67; 194.63] minutes) than natural recesses that followed an earlier natural recess (141.26 [137.74; 144.86] minutes, *N* = 3,220). This difference was statistically significant for both species (type of recess *F*_1,3,174.49_ = 7.81, *p* < .01), and the point estimates for natural recesses that followed an earlier investigator‐initiated recess were 24% longer than natural recesses that followed an earlier natural recess for mallard (141.43 [104.59; 193.40] versus 114.75 [109.96; 119.19] minutes) and 35% longer than natural recesses that followed an earlier natural recess for gadwall (161.18 [134.21; 193.97] versus 119.05 [112.58; 125.81] minutes. However, the relationship between type of recess and recess duration did not differ significantly among species (species * type of recess *F*_1,3,115.96_ = 0.25, *p = *.62) after controlling for time of day (*F*_1,3,174.49_ = 15.86, *p* < .0005), the interaction between time of day and type of recess (*F*_1,3,174.68_ = 15.52, *p* < .0005), incubation day (*F*_1,2,314.05_ = 0.013, *p* = .91), ambient temperature (*F*_1,3,010.65_ = 878.28, *p* < .0001), and day of year (*F*_1,645.28_ = 36.38, *p* < .0001).

### 3.4 Hen habituation and sensitization to repeated incubation interruption

**Figure 4:**
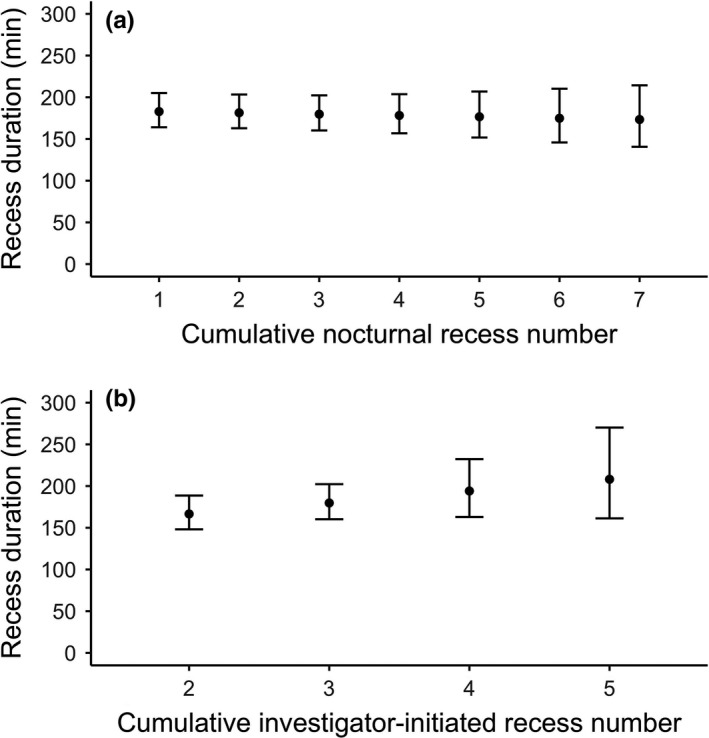
Predicted investigated‐initiated recess duration as a function of (a) the number of previous nocturnal recesses and (b) the number of investigator‐initiated recesses occurring between either nest discovery or incubation onset and that nocturnal recess, for gadwall and mallard at the Grizzly Island Wildlife Area, Suisun Marsh, CA, 2015–2018. Predictions are bootstrapped over 1,000 iterations from a linear mixed model (LMM) which included cumulative investigator flush number, cumulative nocturnal flush number, incubation day, time of day, day of year, and ambient temperature as predictors. Nest identification was included as a random effect. Predictions are shown with time of day held to the mode hour of nest visits and all other non‐focal parameters held to their means. Bars represent 95% prediction intervals

The duration of investigator‐initiated recesses was not influenced by the number of previous nocturnal recesses (*F*_1,301.52_ = 0.36, *p* = .55; Figure 4a) or by the number of previous investigator‐initiated recesses (*F*_1,361.07_ = 2.43, *p* = .12; Figure 4b), after accounting for incubation day (*F*_1,318.93_ = 11.67, *p* < .001), time of day (*F*_1,371.02_ = 28.39, *p* < .0001), day of year *F*_1,307.07_ = 7.57, *p* < .05), and ambient temperature *F*_1,374.86_ = 0.58, *p* = .45).

### 3.5 Effects of hen trapping on incubation recess duration

**Figure 5:**
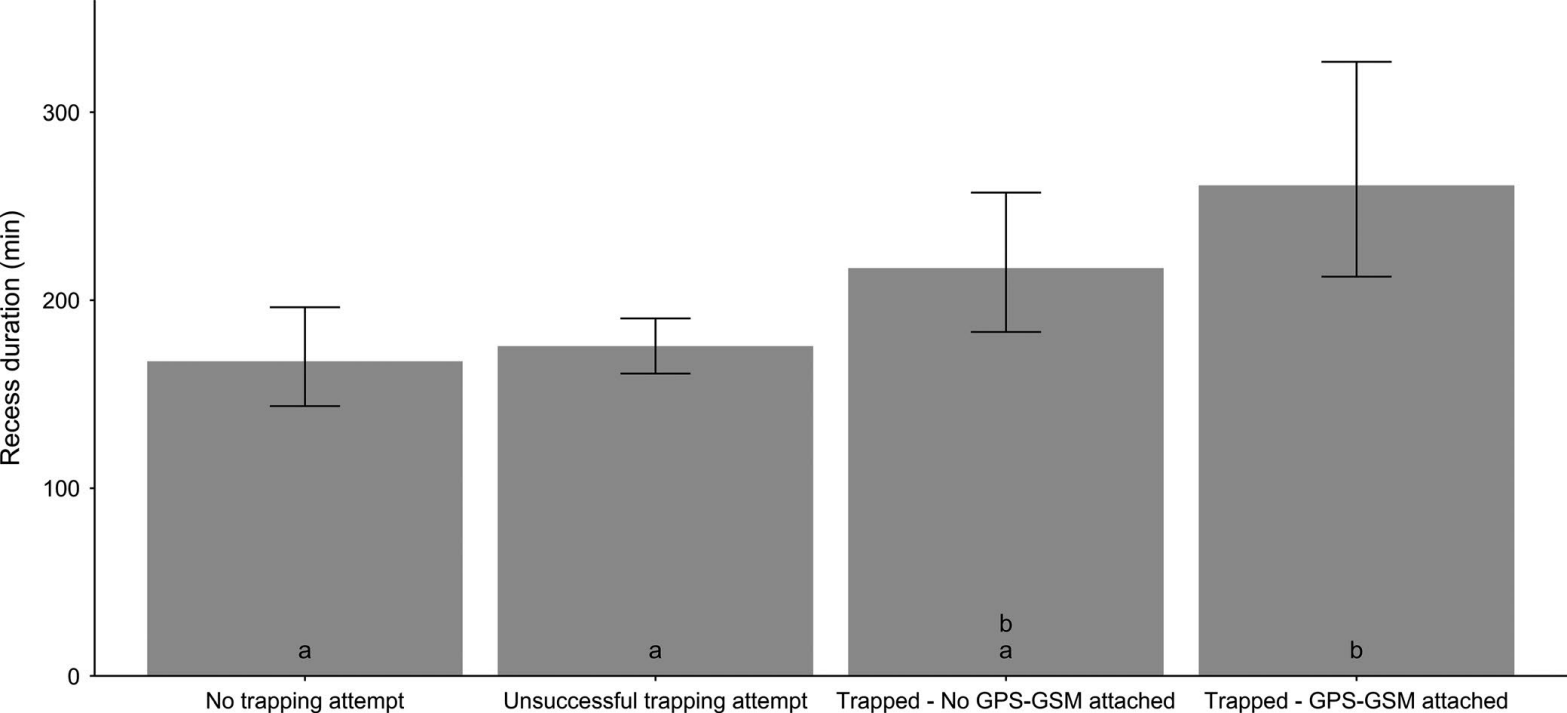
Predicted investigator‐initiated recess duration according to whether trapping was attempted, was successful, and if a transmitter was attached to the hen for mallard and gadwall hens nesting at the Grizzly Island Wildlife Area, Suisun Marsh, CA, 2015–2018. Predictions are bootstrapped over 1,000 iterations from a linear mixed model (LMM) which included type of trapping effort (‘No trapping attempt, ‘Unsuccessful trapping attempt’, ‘Trapped—No GPS‐GSM attached’, ‘Trapped—GPS‐GSM attached’), time of day, and incubation day as fixed effects. Nest identification was included as a random effect. Bars represent 95% prediction intervals. Predictions were generated with time of day held to the mode hour of nest visits and incubation day held at its mean. Bar labels represent significant differences—shared labels indicate no significant difference based on Tukey's post hoc comparisons

We recorded the duration of recesses during 566 investigator visits (including those in which the hen was trapped) at 410 nests. On average, investigator‐initiated recesses (*N* = 68) lasted 219.01 [193.95; 247.32] minutes when investigators did not attempt to trap the hen, 221.17 [206.45; 236.95] minutes when investigators attempted to trap the hen but were unsuccessful (*N* = 403), 275.13 [234.87; 322.28] minutes when investigators successfully trapped hens but did not attach a GPS–GSM transmitter (*N* = 63), and 346.04 [254.52; 470.46] minutes when investigators successfully trapped hens and attached a GPS–GSM transmitter (*N* = 32). Recess duration was longer on average when hens were successfully trapped and handled than when a trapping attempt was unsuccessful or no trapping attempt was made; however recess duration only differed statistically when investigators captured hens and fitted them with GPS–GSM transmitters (*F*_3,527.97_ = 6.57, *p* < .0001; Figure 5). Investigator‐initiated recesses with a successful trapping attempt and a GPS–GSM transmitter attached were 21% longer than investigator‐initiated recesses with a successful trapping attempt but no GPS–GSM transmitter attached, 49% longer than investigator‐initiated recesses with an unsuccessful trapping attempt, and 58% longer than investigator‐initiated recesses with no trapping attempt, after controlling for incubation day (*F*_1,558.58_ = 14.42, *p* < .0001), and time of day (*F*_1,538.72_ = 89.17, *p* < .0001).

## 4 DISCUSSION

Nesting birds must cope with interruptions to incubation caused by predators or other animals moving around the landscape. These interruptions can alter incubation patterns, which can influence overall nest attendance patterns, alter the risk of nest depredation, and ultimately affect reproductive success. Our results demonstrate that mallard and gadwall hens behave differently when incubation recesses are prompted by predator or investigator interruptions versus when hens initiate recesses themselves. When flushed from their nests at night, typically due to predator activity, hens took shorter recesses (23%) than when they initiated recesses themselves during the day. Conversely, daytime recesses that were prompted by investigators were 59% longer than daytime recesses initiated by the hen. There was no difference in the distance travelled between natural diurnal recesses and investigator‐initiated diurnal recesses, but hens travelled farther from their nests during daytime recesses (either natural or investigator‐initiated) than during nocturnal recesses and were more likely to travel to wetland habitats during daytime recesses and upland habitats during nocturnal recesses. Additionally, hens were more likely to take another natural recess if an earlier recess was initiated by investigators, and natural recesses that followed an investigator‐initiated recess earlier in the same day were longer than natural recesses that followed an earlier natural recess. Repeated interruption of incubation, whether by predators or investigators, did not influence the duration of subsequent investigator‐initiated recesses. Recess duration was also not influenced by investigator trapping activity, except when hens were captured by investigators and fitted with GPS–GSM transmitters. In these cases, hens remained away from the nests for longer periods of time after they were released.

The duration of natural recesses differed from that of nocturnal recesses, nocturnal depredation recesses, and investigator‐initiated recesses for both mallard and gadwall. Nocturnal recesses and nocturnal depredation recesses were shorter than natural recesses, whereas investigator‐initiated recesses were longer. That hens returned to nests more quickly at night than during the day may indicate that hens were monitoring events at or near nests after they were flushed by potential predators at night, and/or were acting in defense of their nests by returning more quickly when flushed by a nocturnal predator. Hens may also be acting to mitigate egg‐cooling by returning to nests more quickly under the cooler ambient conditions occurring at night. In addition, nocturnal depredation recesses, where partial clutch depredation is likely to have occurred, did not differ in duration from nocturnal recesses in which partial depredation did not occur. If we assume that in both cases a hen had left the nest because some potential predator was nearby, this suggests that a hen's return to incubation following a nocturnal interruption relates to the cause of the recess rather than the condition of the nest upon return. This is consistent with our earlier studies which revealed that the duration of recesses differed when hens were flushed by different types of predators (Croston, Ackerman, et al., 2018). For example, hens took on average 239 min to return to their nests after raccoons flushed them from their nests, whereas hens returned to nests on average within 81 min when striped skunks flushed them from their nests (Croston, Ackerman, et al., 2018). Trumpeter swan (*Cygnus buccinator*) hens also remained away from their nests for longer periods of time when recesses were triggered by a disturbance than when hens initiated recesses on their own, and responded differently to different stimuli approaching the nest (Henson & Grant, 1991).

We did not find evidence that the maximum distance hens travelled from the nest varied by recess type; however, hens were most likely to use wetland habitat during natural recesses and investigator‐initiated recesses and least likely to use wetland habitat during nocturnal recesses. This finding suggests that incubating hens are less likely to forage (in wetland) when they are flushed from their nests at night than during any recess during the day, but may have been foraging in wetlands during investigator‐initiated recesses. A similar result was observed in trumpeter swans, which spent significantly less time feeding and preening when leaving nests due to a disturbance than under normal recess conditions (Henson & Grant, 1991). Dabbling duck hens in Suisun Marsh were also more likely to take an additional recess following an investigator‐initiated recess the same day than following a natural recess. These results together suggest that when incubation is interrupted hens may not meet their daily self‐maintenance needs as they would during a natural recess, often necessitating that the hen take another recess later in the day. Thus, incubation interruptions likely increase the time hens spend off the nest with less benefit to the hen during the recess, and may increase the length of the overall incubation period and in turn increase the risk of depredation.

Repeated interruptions during incubation over several days and weeks, whether by predators or investigators, did not influence the duration of subsequent investigator‐initiated recesses. This suggests that hens neither habituated nor became sensitized to repeated contact with either investigators or predators over the course of our study. Previous studies of both mallard and gadwall have shown sensitization to repeated investigator disturbance as measured by flushing distance (Gunness & Weatherhead, 2002); however, this behavior may not correlate positively with recess duration (Mallory & Weatherhead, 1993). Likewise, common goldeneyes *Bucephala clangula* that were repeatedly visited by investigators did not take longer to return to their nests following a visit by investigators but did increase nest defense behaviors with repeated visits (Mallory & Weatherhead, 1993). Habituation has been demonstrated in several waterfowl and waterbird species (e.g. Baudains & Lloyd, 2007; Vennesland, 2010), but lack of habituation has also been observed (e.g. Conomy et al., 1998).

There also was no difference in recess duration among recesses that were initiated when investigators approached nests but did not try to trap hens, or when investigators either successfully or unsuccessfully attempted to trap hens. However, hens remained away from nests for longer periods of time when they were captured on nests and fitted with GPS–GSM transmitters. This suggests that wearing the transmitter delayed the hens’ return to their nests, possibly because they spent time examining and preening around the transmitter once they were released (Barron et al., 2010). For example, mallards wearing backpack transmitters spent less time feeding and more time resting and preening than mallards not wearing these devices (Gilmer et al., 1974, Pietz et al. 1993), and abnormal behavior after transmitter attachment occurs regularly in waterfowl (e.g. Perry, 1981, Garrettson et al., 2000, Kesler et al., 2014; reviewed in Calvo & Furness, 1992 and Barron et al., 2010). Researchers can minimize these effects by continuing to advance the design of wearable tracking devices to reduce interference with hen mobility. In addition, attaching transmitters earlier in the day would likely decrease the risk of hens remaining away from their nests overnight when transmitters are attached (see Croston et al., 2020).

While predator and investigator‐initiated incubation interruptions had a measurable effect on dabbling duck hen incubation recesses, these effects seem to be limited and specific to both the nature of the interruption and the time of day when it occurs.

## CONFLICT OF INTEREST

The authors declare no conflict of interest.

## AUTHOR CONTRIBUTION

**Rebecca Croston:** Conceptualization (equal); Data curation (equal); Formal analysis (lead); Methodology (equal); Visualization (lead); Writing‐original draft (lead); Writing‐review & editing (equal). **C. Alex Hartman:** Conceptualization (equal); Data curation (equal); Formal analysis (equal); Methodology (equal); Validation (equal); Visualization (equal); Writing‐original draft (equal); Writing‐review & editing (equal). **Mark P Herzog:** Conceptualization (equal); Data curation (equal); Formal analysis (equal); Methodology (equal); Validation (equal); Visualization (equal); Writing‐review & editing (equal). **Sarah H Peterson:** Conceptualization (equal); Data curation (equal); Formal analysis (equal); Methodology (equal); Validation (equal); Visualization (equal); Writing‐review & editing (equal). **Jeffrey D Kohl:** Data curation (equal); Methodology (supporting). **Cory T Overton:** Data curation (supporting); Methodology (supporting). **Cliff L Feldheim:** Project administration (equal); Resources (equal). **Michael L Casazza:** Data curation (equal); Project administration (equal); Resources (equal). **Joshua T Ackerman:** Conceptualization (equal); Data curation (equal); Formal analysis (equal); Methodology (equal); Project administration (equal); Resources (equal); Supervision (equal); Validation (equal); Visualization (equal); Writing‐original draft (equal); Writing‐review & editing (equal).

## ETHICAL APPROVAL

Research was conducted with the approval of the U.S. Geological Survey Western Ecological Research Center's Animal Care and Use Committee.

## Data Availability

The raw data in this manuscript are available via ScienceBase (https://doi.org/10.5066/P9JXF6J3).
